# Development of genetic evaluation for milk production traits of Holsteins in Japan

**DOI:** 10.1111/asj.13190

**Published:** 2019-02-14

**Authors:** Koichi Hagiya

**Affiliations:** ^1^ Department of Life and Food Science Obihiro University of Agriculture and Veterinary Medicine Obihiro Japan

**Keywords:** dairy cattle, genetic evaluation, heterogeneity within herd variance, Holstein, test‐day model

## Abstract

The procedure used for the genetic evaluation of dairy cattle in Japan has developed from a lactation sire–MGS model to a multiple‐lactation random regression test‐day animal model. Genetic evaluation of Holstein bulls in Japan began in 1989 with the use of field‐style progeny testing; dairy herd improvement program data from all over Japan were used, along with a sire and maternal grandsire model. In 1993, an animal model was introduced to estimate breeding values for yield and type traits. A random regression test‐day model was first applied in 2010. In the business of breeding dairy cattle, it is very important to users that estimated breeding values are reliable and stable among subsequent routine evaluations. With experience in the genetic evaluation of dairy cattle in Japan, Japanese researchers have found ways to improve the stability of estimated breeding values. These modifications involve changes in data editing, development of evaluation models, changes to the structures of unknown‐parent groups, awareness of the problems of predicting lactation yield from partial test‐day records, and adjustment for heterogeneity within herd variances. Here, I introduce developments in, and our experiences with, the genetic evaluation of yield traits of Holstein cattle in Japan.

## 1 INTRODUCTION

In Japan, 99% of dairy cattle are Holsteins. Genetic evaluation of Holstein bulls began to be published in 1989 with the use of field‐style progeny testing (PT); dairy herd improvement (DHI) program data from all over Japan were used, along with a sire and maternal grandsire (MGS) model (National Livestock Breeding Center, 1999). Estimated transmitting abilities were obtained for yield traits, including milk, fat, protein, and solid nonfat (SNF) yields, as well as fat, protein, and SNF percentages, in cow's milk. Sires were ranked according to an economic index calculated by using milk prices.

In 1993, an animal model was introduced to estimate breeding values for yield and type traits (National Livestock Breeding Center, 1993). Details of the top cows in Japan, as ranked by the economic index, were published in the same way as for the bulls. Farmers and artificial insemination (AI) technicians became able to select bulls by using the estimated breeding values (EBVs) of yield traits and conformation traits (Table 1). Management traits—milking speed, temperament, and calving ease—began to be evaluated and published in 1997 (Table 1). These traits were estimated by using a threshold sire–MGS model. In 1998, bulls were ranked for the first time by the Nippon total profit index (NTP) as a total merit index. The first NTP contained EBVs for fat and protein contents and for conformation traits such as mammary system, udder depth, and fore‐udder attachment.

**Table 1:** Timeline of the introduction of genetic evaluations in Japan and related events

Year	Event
1993	Animal model for production and conformation traits
1997	Liability sire and maternal grandsire (MGS) model for temperament, milking speed, and calving ease
2003	Animal model for somatic cell score and adjustment for heterogeneity of herd variance for yield traits
2006	Multiple‐trait animal model for herd life
2008	Animal model for lactation persistency
2010	Random regression repeatability test‐day model for yield traits and lactation persistency
2011	Liability sire and MGS model for stillbirth
2013	Genomic enhanced breeding value (GEBV) published for heifers
2014	Animal model for conception rate and days open
2015	Multiple‐lactation random regression test‐day model for yields and lactation persistency
2017	GEBV for bulls and cows

An EBV for somatic cell score was published in 2003. At the same time, bulls in Japan attended a MACE (multiple‐trait across‐country evaluation) conducted by INTERBULL (Interbull, 2018). In 2006, herd life published as a longevity trait (Hagiya et al., 2012), and in 2008 a model that included lactation persistency (LP) as a new health trait was published (Table 1). The EBV of herd life was estimated by using a multiple‐trait animal model. Multiple‐trait prediction (Schaeffer & Jamrozik, 1996) was used to estimate LP for each lactation, and a single‐trait animal model was used to estimate the EBV of LP. A random regression test‐day model (RR‐TDM) was first applied to yield traits in 2010. The estimation of LP was then updated by using the RR‐TDM. Stillbirth modeling began to be published in 2011 (Table 1). The EBV of conception rate and days open associated with female fertility traits were published in 2014 (Atagi & Hagiya, 2005; Hagiya et al., 2014). The RR‐TDM was updated to a multiple‐lactation model in 2015. Genomic EBVs were published in Japan for heifers in 2013 and for bulls in 2017.

Here, I introduce the developments in, and our experiences of, the genetic evaluation of yield traits in Holstein cattle in Japan.

## 2 DATA COLLECTION SYSTEM

Most dairy cattle in Japan are bred through AI using frozen semen. In 1969, PT was started in 180 young Holstein bulls in Japan to evaluate dairy bulls’ genetic performance on the basis of their daughters’ records (National Livestock Breeding Center (NLBC), 1993). Daughters of the bulls were tested for milk, fat, protein, and SNF yields at PT stations run by the National Livestock Breeding Station. The Japanese system of PT differed from those in other countries because in Japan the testing stations were run by the government. This system of PT was the only choice then available because no DHI program existed at the time in Japan (Abe, 1993). Selected bulls were used throughout Japan through AI. Genetic evaluations of bulls were made by using herdmate comparisons (Mitsumoto, 1980). However, PT stations had the disadvantage of being expensive, and testing facilities were therefore limited (Touchberry, Rottensten, & Andersen, 1959).

Japan's DHI program started in 1974. The traditional DHI collected monthly records of milk production, milk fat, milk protein, and SNF yields and percentages, along with such characteristics as animal ID, birth date, calving date, parity, and days in milk (DIM). The DHI program service has expanded over the years, and the number of licensed herds and cows has increased. In 1984, by which time about 34% of all cows were DHI licensed (Livestock Improvement Association of Japan, 2018), new PT using farmers’ herds—called field testing—was introduced in Japan. As part of this field testing, bull semen was distributed to licensed DHI dairy farms throughout Japan. Daughters of PT bulls were produced in farmers’ herds and their data recorded with those of their contemporaries from calving to at least 240 DIM. For the first few years, data on daughters were collected from both PT stations and farmers’ herds, but collection gradually shifted toward field testing.

Classification records were collected from daughters in herds participating in PT. The conformation traits of bulls’ daughters and their contemporaries were recorded by professional classifiers from the Holstein Association of Japan. Conformation traits were evaluated by using the recommended standard linear traits and definitions of type traits published by the World Holstein Friesian Federation (2016).

Genetic evaluation of Holstein bulls using records from all over Japan began in 1989 by using a sire–MGS model, with data from DHI, and classification and pedigree records from the Holstein Association of Japan. Thereafter, AI bulls were generally selected by genetic evaluation.

## 3 LACTATION MODEL

In 1993, the first EBVs in Japan of 2.1 million dairy cattle were estimated by using an animal model (Abe, 1993). The EBVs were published for all PT bulls and top‐ranked cows. Data contained in the animal model for milk yield traits were records of milk yields and conformation traits on Holstein cows aged from 22 to 35 months at first calving, and lactation records from the first to fifth parities, obtained from two milkings a day. The sum of the daily milk yields from calving to 305 DIM was calculated for each cow as the 305‐day lactation yield. When a cow had test‐day records for fewer than 305 DIM, the lactation records were expanded from monthly milk records to a 305‐day yield by using Method P (Miller, Pearson, Fohrman, & Creegan, 1972). A record was treated as a missing value when the lactation period finished with fewer than 240 DIM (National Livestock Breeding Center, 1993). EBV was estimated by using a single‐trait animal model, as follows:

(1) $$y_{\mathrm{ijklmn}}={\text{HY}}_{i}+C_{j}+M_{k}+A_{l}+u_{m}+{\text{pe}}_{m}+e_{\mathrm{ijklmn}},$$

where *y*_*ijklmn*_ is lactation yield, HY_*i*_ is the fixed effect of herd‐year *i*,* C*_*j*_ is the fixed effect of country *j* of the cow's bull, *M*_*k*_ is the fixed effect of calving month *k* (12 calendar months) within area (Hokkaido or Honshu), *A*_*l*_ is the fixed effect of calving age *l*,* u*_*m*_ is the random additive genetic effect of animal *m*, pe_*m*_ is the random permanent environmental effect on animal *m*, and *e*_*ijklmn*_ is the random residual effect associated with lactation yield. In the early 1990s, frozen semen imported from foreign countries was generally very expensive and tended to be used only on superior cows or heifers. The effect of bull's country represents the effect of cow selection in the case of cows mated by using imported frozen semen. The impact of the first EBVs estimated for cows by using the above animal model was great, and we found many new, superior lines.

The statistical model for yield traits was changed in 1996, as follows (National Livestock Breeding Center, 1996):

(2) $$y_{\mathrm{ijkl}}={\text{HYP}}_{i}+M_{j}+u_{k}+{\text{pe}}_{k}+e_{\mathrm{ijkl}}$$

where *y*_*ijkl*_ is the lactation yield preadjusted for the effect of parity and age at calving, HYP_*i*_ is the fixed effect of herd‐year parity *i*, and *M*_*j*_, $u_{k}$, pe_*k*_, and *e*_*ijkl*_ are the same as in Equation (1). This model worked well, but in 1999 it was modified to include a term related to year effect (National Livestock Breeding Center, 1999):

(3) $$y_{\mathrm{ijkl}}={\text{HYP}}_{i}+{\text{MY}}_{j}+u_{k}+{\text{pe}}_{k}+e_{\mathrm{ijkl}}$$

where MY_*j*_ is the fixed effect of month‐year *j* and the other terms are the same as in Equation (2). This modification accounted for the differences in seasonal effects from year to year. In this model, year effects were included in both HYP_*i*_ and MY_*j*_; therefore, the total effect, as shown by HYP_*i*_ + MY_*j*_, was stable among subsequent routine evaluations. However, the estimated effects of each of HYP_*i*_ and MY_*j*_ separately sometimes differed among routine evaluations, and this caused confusion in the description of the fixed effect. This problem suggested that a simple model would be preferable for routine genetic evaluation in dairy cattle.

## 4 IMPACT OF THE DROP IN BULL EBVS

In the early 1990s, semen from one of the highest‐ranking bulls in Canada, Ronnybrook Prelude ET (HOCANM0000392457), born in 1986, was used for AI worldwide. In the business of breeding dairy cattle, the substantial drop in this bull's EBVs was a shocking fact (Lohuis & Schaeffer, 1995). Also, in Japan, bull EBVs sometimes changed considerably between two subsequent routine evaluations. We found three reasons as to why a bull's EBV stability could be compromised.

The first reason why two subsequent EBVs differ from each other was related to data editing. When a cow with fewer than 305 DIM was still in milk, her lactation yield was estimated by using test‐day yields and was included in the genetic evaluation. However, when a cow's lactation finished after fewer than 240 DIM, her lactation record was deleted from the files used for the genetic evaluation. The EBV of the bull changed when his daughter's records, which had been used in the previous genetic evaluation, were then deleted from the current genetic evaluation. This situation should have been avoided in our data editing.

Second, we used genetic groups (Quaas & Pollak, 1981) to represent unknown‐parent groups (UPGs) of animals in the pedigree to account for genetic trends. Group solutions represent the average EBVs of unknown (unidentified or represented by only one descendant) animals selected to be parents without records (Westell, Wuaas, & Van Vleck, 1988). We made phantom parent groups (i.e., UPGs) according to birth year, as estimated by using those of the progeny. For example, we assumed that UPGs were made up of groups of animals from younger to older and contained an unknown parent every 5 years. The UPG for younger animals therefore contained the most recent unknown parents (i.e., those within the 5 most recent years). The members of the youngest UPG changed from year to year. The EBV estimates of a bull that had only a few daughters were thus affected when the unknown parents in his pedigree changed. This is the second reason why two subsequent EBVs could change. We learned that we should therefore not change the members of the current UPG and those of the previous UPG. In other words, UPG members should be fixed based on animal's birth year.

The third reason was prediction error, caused mainly by the prediction of lactation yields from partial test‐day records in early DIM. Method P can predict future yields by using the latest test‐day records and assumes a standard lactation curve (Miller et al., 1972). In other words, it cannot adjust for differences in the shapes of the lactation curves of particular individual cows. Therefore, when a bull had extremely high or low lactation persistency, the estimated lactation yield was not similar to the observed yield. The lactation yields estimated by multiple‐trait prediction (Schaeffer & Jamrozik, 1996) or best prediction (VanRaden, 1997) are better than those of method P, however, problems still might not be solved completely. In the case of the lactation model, records of lactation yield were used, therefore, it would be difficult to avoid this problem. The remaining problems associated with the prediction of lactation yields were solved when an RR‐TDM was used.

## 5 RANDOM REGRESSION TEST‐DAY MODEL

A practical test‐day model using a random regression in dairy cattle was introduced by Schaeffer, Jamrozik, and Dekkers (1994). In Canada, official EBVs were estimated for the first time in the world by using an RR‐TDM, published in 1999, and were used to replace EBVs determined by using an animal lactation model (Schaeffer, Jamrozik, Kistemaker, & Van Doormaal, 2000). The RR‐TDM was superior from both a theoretical and a practical perspective. For example, it could use all test‐day records without predicting lactation yields, and it could consider various lactation curves for each cow. In the 2000s, many countries followed Canada's lead and introduced the RR‐TDM.

In 2010, we introduced a repeatability RR‐TDM introduced in Japan as follows:

$$y_{\mathrm{ijkl}}={\left({\text{HTD}}_{i}+\sum_{m\;=\;0}^{5}b_{\mathrm{jm}}w{\left(t\right)}_{\mathrm{klm}}+\sum_{n\;=\;0}^{2}u_{\mathrm{kn}}z{\left(t\right)}_{\mathrm{kln}}+\sum_{n\;=\;0}^{2}p_{\mathrm{kn}}z{\left(t\right)}_{\mathrm{kln}}+e_{\mathrm{ijkl}}\right)}^{\exp(\gamma_{i}/2)},$$

where *y*_*ijkl*_ is test‐day yield, HTD_*i*_ is the fixed effect of herd test‐day *i*,* b*_*jm*_ is the *m* th fixed regression coefficient specific to subclass *j* of the region (Hokkaido and Honshu)—calendar month—age group at calving, *u*_*kn*_ and *p*_*kn*_ are the *n* th random regression coefficients specific to cow *k* for additive genetic and permanent environmental effects, respectively, *w*(*t*)_*klm*_ and *z*(*t*)_*klm*_ are covariates for fixed and random regressions, respectively, associated with DIM *t* for test‐day record *l* of cow *k*(*t*_*kl*_), and *e*_*ijkl*_ is the random residuals associated with each record. The covariates of regression are fourth‐order Legendre polynomials with the exponential term of the Wilmink function (Schaeffer et al., 2000) for the fixed lactation curves and second‐order Legendre polynomials for random terms (Kistemaker, 2003). γ_*i*_ takes into account the autoregressive model (Kachman & Everett, 1993).

**Figure 1:**
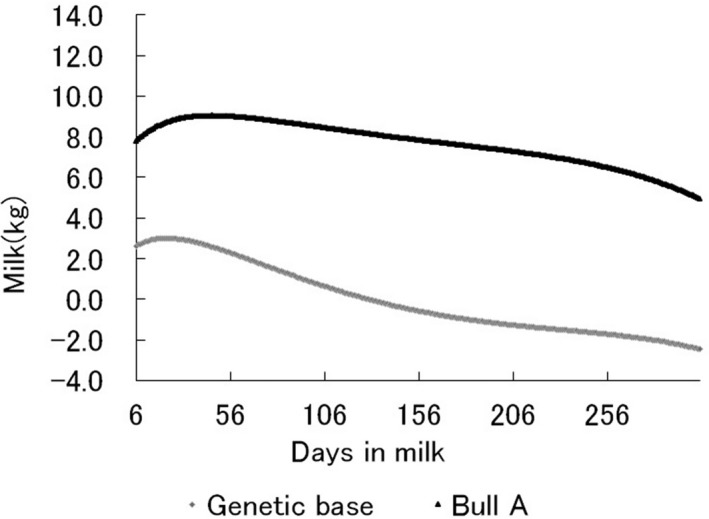
An example of the genetic lactation curve of a bull and lactation curve for genetic base

The new RR‐TDM avoided the problem of extension from test‐day record to lactation record. Moreover, additional information such as lactation persistency could now be calculated easily (Togashi et al., 2008). In Japan, the second‐order Legendre polynomial was used to show genetic lactation curves for bulls (Figure 1). In many countries, in contrast, third‐ or fourth‐order polynomial functions are used to estimate genetic lactation curves for particular animals in RR‐TDMs (Interbull, 2018). In our preliminary analysis, we found that a quadratic polynomial was preferable to the others in showing the genetic lactation curves of bulls.

## 6 ADJUSTMENT FOR HETEROGENEITY WITHIN HERD VARIANCE

Heterogeneity of genetic and residual variances within herds exists for milk production and other traits (e.g., De Veer & Van Vleck, 1987; Everett, Keown, & Taylor, 1982). Variance components of milk yield have been estimated from herds grouped by production level, revealing a positive correlation between production level and these variance components (Boldman & Freeman, 1990). Everett et al. (1982) proposed a method of adjusting for heterogeneous phenotypic variances across contemporary animals by applying a log transformation. A procedure for adjustment of heterogeneous phenotypic variances was also developed by using an empirical Bayes method (Weigel & Gianola, 1992; Wiggans & VanRaden, 1991). When the heterogeneity is not adjusted in a genetic evaluation, differences within herd subclass variances result in biased EBV estimates (Weigel & Gianola, 1992). Meuwissen, De Jong, and Engel (1996) reported a method of estimating breeding values and correcting for heterogeneous phenotypic variances by applying an autoregressive model. Their procedure considered covariance across genetic relationships and the reduction in variance caused by selection. In 2003, adjustment of heterogeneous variances by using the autoregressive model of Meuwissen et al. was applied to a lactation animal model in Japan (Hagiya, Atagi, Shirai, & Suzuki, 2005):

$$y_{\mathrm{ijklm}}={\left({\text{HYPF}}_{i}+{\text{RMY}}_{j}+A_{k}+u_{l}+p_{l}+e_{\mathrm{ijklm}}\right)}^{\exp(\gamma_{i}/2)},$$

where *y*_*ijklm*_ is lactation yield, HYPF_*i*_ is the fixed effect of herd—year—parity—milking frequency in a day *i*, RMY_*j*_ is the fixed effect of region‐calving month‐calving year, *A*_*k*_ is the fixed effect of age group at calving *k*,* u*_*l*_ and *p*_*l*_ are random effects for cow *l* for additive genetic and permanent environmental effects, respectively, and *e*_*ijklm*_ is the random residuals associated with each record. γ_*i*_ takes into account an autoregressive model containing fixed and random effects (Kachman & Everett, 1993). This autoregressive model was used even after the evaluation method was changed to a RR‐TDM.

The use of an autoregressive model in our genetic evaluation seems to be more appropriate than preadjustment from the perspective of theoretical prediction. When the autoregressive model was applied to our genetic evaluations, the estimated values of γ_*i*_ were expected to be close to 1.0. However, they were sometimes far from 1 when the herd size was small. Therefore, the range of possible values of γ_*i*_ was restricted—for example, ranging from 0.5 to 2.0. In addition, when the average value of γ_*i*_ was larger (or smaller) than 1, the genetic and phenotypic trends tended to have excessively high or low values. The procedure for adjusting heterogeneous phenotypic variances was changed to preadjustment (Kistemaker & Schaeffer, 1998) in 2015. If the autoregressive model was to be reintroduced, we would have to control the average γ_*i*_ value to make it equal to 1.

## 7 CONCLUSIONS

The procedure used for the genetic evaluation of dairy cattle in Japan has developed from a lactation sire–MGS model to a multiple‐lactation RR‐TDM. The data collection system has also developed in response to the efforts of those involved. In genetic evaluation in Japan, we need to improve the stability of EBVs between two subsequent routine evaluations; that is, we need to improve the durability of the model, further develop the data collection system, and learn from past failures. I hope that this review will help those who have just begun to work in our genetic evaluation system, as well as young researchers in the field of dairy cattle breeding.
